# Research on N_2_-inhibitor-water mist fire prevention and extinguishing technology and equipment in coal mine goaf

**DOI:** 10.1371/journal.pone.0222003

**Published:** 2019-09-04

**Authors:** Hongwei Liu, Fei Wang

**Affiliations:** 1 College of Safety and Emergency Management Engineering, Taiyuan University of Technology, Taiyuan, China; 2 Center of Shanxi Mine Safety for Graduate Education Innovation, Taiyuan, China; China University of Mining and Technology, CHINA

## Abstract

In this study, a new type of N_2_-inhibitor-water mist (NIWM) technology was proposed to resolve the problem of fire prevention and extinguishing in the goaf of coal mine. The corresponding equipment was designed and manufactured. Under the condition that both gas pressure and liquid pressure were 0.5–2MPa, the NIWM equipment produced the water mist with Sauter mean diameter (SMD) range of 166–265μm. The experimental results of the operating parameters of NIWM equipment were in agreement with the theoretical derivation. The theory of two-phase flow atomisation can be used as theoretical guide for this technology. After that, on the basis of the NIWM equipment, the experiments of inhibiting low temperature (30–100°C) oxidation and extinguishing high temperature combustion of large dosage of coal sample were carried out. Water mist with SMD = 188μm had good diffusivity in the container. The inhibiting effect of N_2_-inhibitor-water mist on low temperature oxidation of coal was obviously greater than that of single material. N_2_-water mist extinguished the burning coal completely in 20 minutes. The addition of water mist solved the shortcoming of poor cooling effect of N_2_. In different stages of coal-oxygen reaction, N_2_, inhibitor and water mist play very different role in controlling the process of coal-oxygen reaction, which was not simple accumulation of the three. The combination of N_2_, inhibitor and water mist should be determined according to the state of the coal mine goaf fire. On the basis of the research conclusions, the onsite arrangement diagram of the NIWM fire prevention and extinguishing equipment in the goaf was designed. The research results proved the feasibility and effectiveness of this technology, and it is of great significance to the prevention and control of coal spontaneous combustion in goaf.

## Introduction

Spontaneous combustion of coal mine is the fire occurred in underground coal seam. These fires occupy a large area, exhibit high temperatures, and continue for a long duration. The spontaneous combustion of coal has been reported in major coal producing countries such as the United States, Canada, China, Australia, India, Indonesia, South Africa, Britain, Germany, Poland, Czech Republic, Russia, Ukraine, Turkey, Thailand, Colombia, Egypt, France, Portugal, and New Zealand. Spontaneous combustion of coal is more serious in China, India, the United States, and South Africa[1]. It can deplete a significant amount of coal resources. In China, a maximum of 20 million tons of coal was estimated to be burned each year[2]. The spontaneous combustion of coal can lead to the release of large volumes of greenhouse gases, such as CO_2_ and methane, and toxic gases, along with dangerous health hazards that seriously threaten local residents[3]. Volatile elements such as arsenic, fluorine, and mercury are generally enriched in coal[4]. With the combustion of coal, these elements can be volatilised and eventually absorbed by humans and animals[5]. In addition, spontaneous combustion of coal can lead to gas and coal dust explosions in mines, which seriously threatens the safety of mine production and staff[6–8].

China is the largest coal producing and consuming country in the world. In 2018, the output of raw coal in the world was 8.013 billion tons, and China’s coal production was 3.683 billion tons, accounting for 46% of the total output of the world. More than 50% of China’s coal mines are in danger of spontaneous combustion, and fires in the goaf account for 60% of the spontaneous combustion of coal[9]. China’s coal mine fires have important impact on global greenhouse gas emissions[10].

At present, the main technologies for controlling fires in the goaf include water injection, plugging, pressure equalising, grouting[11], inhibitor[12], inert gas[13], gel[14], and three-phase foam[15,16]. The above-mentioned technologies all have their own specific advantages. They have been widely applied in fire prevention and extinguishing practices and have produced remarkable social and economic benefits[9]. However, they also face problems to various degrees, and it is difficult to achieve good results in terms of both their control effectiveness and their economic benefit. Table 1 shows the advantages and disadvantages of the common fire prevention and extinguishing technologies in coal mine.

**Table 1:** Advantages and disadvantages of the common fire prevention and extinguishing technologies in coal mine.

Fire prevention technology	Main material	Advantages	Disadvantages	Cost(RMB/m^3^)
Grouting	Yellow mud, sand, gangue, fly ash, cement mortar	(1) Wrap the coal body, isolate the contact between coal and oxygen; (2) Endothermic and cooling; (3) Simple process and low cost.	(1) Stacking property is poor; (2) Slurry uneven covered; (3) Easy to run and pulp, affect coal quality, deteriorate working environment.	10~30
Pressure equalising	Underground ventilation facilities	(1) Control oxygen supply at source; (2) Remarkable effect; (3) Do not pollute the environment.	(1) Complex operation; (2) Poor stability; (3) High requirements for personnel quality.	Low
Water	Tap or mine water	(1) The endothermic and cooling rate is fast; (2) Reduce the temperature of fire surface, reduce oxygen concentration, and inert the fire area; (3) Low cost.	(1) Strong fluidity, small coverage area, difficult to stay at high places; (2) Water is easy to run, deteriorate the underground environment; (3) Increase air leakage.	Low
Inhibitor	NaCl, KCl, MgCl_2_, CaCl_2_ and organic substances	(1) Prevent coal oxidation by making coal body surface active structure inactive; (2) Absorb heat and reduce temperature, and keep the coal in a moist state for a long time.	(1) Not easy to disperse evenly on the coal body, and the spraying process is difficult to implement; (2) Corrode underground equipment and affect the health of underground workers.	30~50
Inert gas	N_2_, CO_2_ and other inert gases	(1) Reduce the oxygen concentration in the area; (2) Make gas and other combustible gases lose explosiveness in the fire area; (3) No corrosion to equipment, and does not affect the health of workers.	(1) Easy to diffuse with the air leakage, and not easy to stay in the injected area; (2) The injection machine needs constant maintenance; (3) Poor cooling and extinguishing effect.	Low
Plugging	Rocsil, high water quick setting material, horsepower scattered	(1) Has good pressure resistance and plugging effect; (2) Isolate oxygen; (3) The effect of preventing air leakage is better.	(1) The workload is large; (2) High cost; (3) Releases harmful gases at high temperatures; (4) Easy to burn at high temperatures.	80~1000
Gel	Ammonium gel	(1) The effect of wrapping coal body and sealing cracks is good; (2) High temperature resistance; (3) Obvious effect on partial fire.	(1) Small flow, poor mobility, and difficult to use in large areas; (2) The gel will crack over time; (3) Produce toxic and harmful gases; (4) High cost.	60~80
	Polymer gel			100~150
Inert gas foam	N_2_ foam, CO_2_ foam	(1)Avoid “pull ditch” phenomenon; (2) Water can be evenly distributed; (3) Suitable for coal mining in deep coal-mining area.	(1) Foam breaks easily; (2) Foam only contains liquid water, and once the water evaporates, the performance of fire prevention and extinguishment will disappear.	Low
NIWM	N_2_, inhibitor, water mist	(1) Comprehensively utilising the fire control characteristics of the N_2_, inhibitor, and water mist; (2) N_2_ is used as the pneumatic atomising-gas source; (3) N_2_ is the carrier of inhibitor-water mist.	(1) Two-phase flow atomisation is involved, and the equipment is more complicated than single-phase flow; (2) Theory is not yet mature, and there are few engineering application examples.	Low

Therefore, it is urgent to propose a more economical and effective fire prevention and extinguishing technology in coal mine goaf. In view of the above situation, this study proposed a N_2_-inhibitor-water mist (NIWM) technology for fire prevention and extinguishing. This technology selected a highly effective inhibitor, and then designed and manufactured a pneumatic inhibitor-water mist generator. In this generator, N_2_ was used as the pneumatic atomising-gas source to atomise the inhibitor liquid. The inhibitor-water mist was transported to every corner of the goaf area through the fluidity and diffusivity of air leakage and N_2_ in the goaf. This process achieved three-dimensional coverage of the high-temperature coal and rock in the goaf by the N_2_, inhibitor, and water mist. This technology can effectively control a goaf fire by comprehensively utilising the fire control characteristics of the N_2_, inhibitor, and water mist. Since this technology involves the atomisation process of the two-phase flow, the equipment is relatively complex compared with single-phase flow fire prevention and extinguishing technology. Therefore, it is necessary to design corresponding equipment, and at the same time determine the operating parameters of the equipment. In addition, whether the fire prevention and extinguishing effect of NIWM can achieve the cumulative effect of the three technologies requires further experimental verification. Thus, in this paper, the NIWM equipment was designed and manufactured, and the operating parameters of the equipment were theoretically analyzed and experimentally verified. Then, the experimental study of inhibiting low temperature oxidation of coal and extinguishing combustion of coal was carried out by using designed NIWM equipment. The research results of this paper can provide theoretical basis for the application of this technology.

## NIWM equipment design and operation

### NIWM equipment design

**Fig 1:**
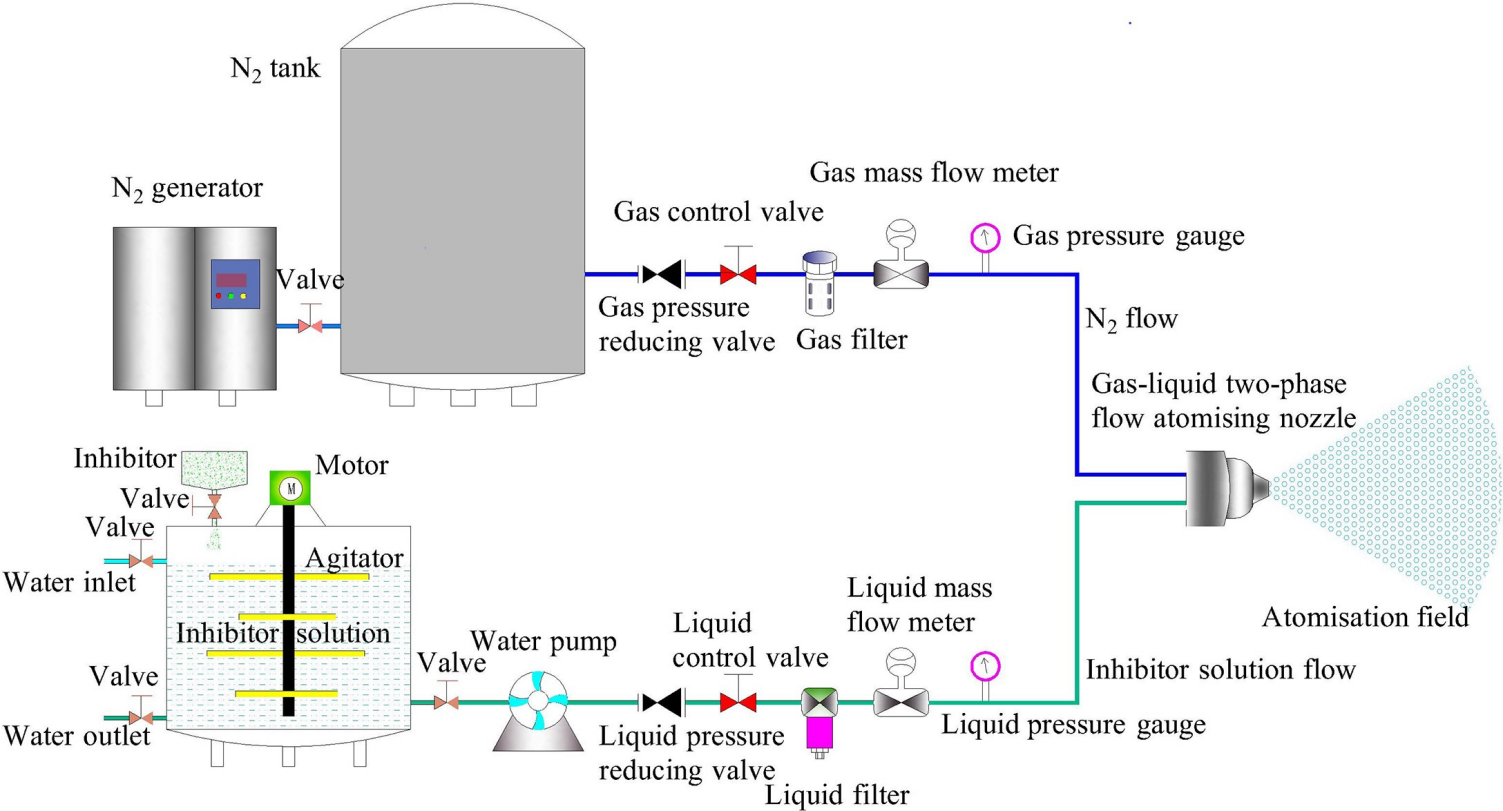
Design of NIWM goaf fire prevention and extinguishing equipment.

**Fig 2:**
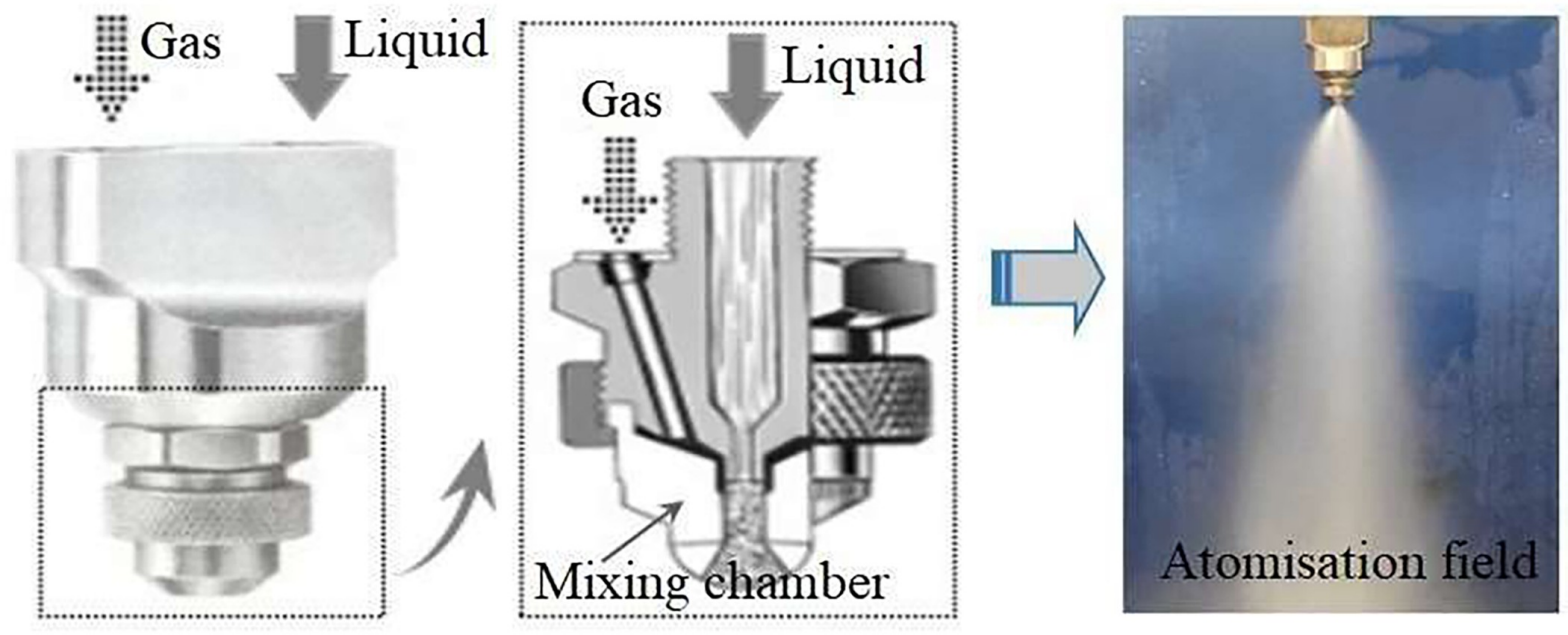
Internal structure and atomisation effect of the gas–liquid two–phase flow atomising nozzle.

The water mist spray technology is diverse and increasingly mature, and its application range is gradually expanding. However, from the principle of its production, the generation of water mist should make full use of the velocity gradient of the atomised liquid and surrounding gas. According to the way in which the spray jet-flow interacts with the surrounding air, it can be classified into the following two types[17]. The first type involves the release of a high-pressure jet-flow through a nozzle into relatively low-speed air; a pressure or rotary type nozzle can be used. In the second type, a low-speed jet-flow is diffused into a high-speed airflow environment; in this case, a two-fluid nozzle of the pneumatic, air-blast, or bubble type can be used. This study proposed to use the two-fluid atomisation technology to achieve the preparation of the two-phase flow of N_2_ and inhibitor-water mist. The design diagram of the NIWM fire prevention and extinguishing equipment for goaf area is shown in Fig 1. According to the different gas-liquid mixing methods, the air-atomising nozzles can be classified into internal and external mixing types. Compared with the external mixing atomising nozzle, the internal mixing atomising nozzle can obtain a good atomisation effect and ideal droplet distribution. Therefore, this study proposed to adopt the internal mixing nozzle for goaf fire prevention and extinguishing. The internal structure and fluid flow of the internal mixing atomising nozzle are complicated, and the main factors affecting the internal flow field and flow characteristics of the nozzle are the nozzle structure and operating parameters. Therefore, it is necessary to study these factors. Fig 2 is the internal structure and atomisation effect of the gas-liquid two-phase flow atomising nozzle.

### NIWM equipment operation

The operation of the equipment in Fig 1 involves the gas/liquid transport process in the pipeline and the two-phase flow atomisation process in the gas-liquid two-phase flow nozzle. The gas/liquid transport process in the NIWM equipment is the flow of a single fluid in the pipeline, the theory is relatively mature, and no further research is conducted here. This paper focused on theoretical derivation and experimental verification of the atomization process of two-phase flow. Because the inhibitor has little effect on the two-phase flow atomisation process, the effect of inhibitor on the atomisation characteristics has not been studied separately.

#### Theoretical derivation of operating parameters of NIWM equipment

As shown in Fig 2, the rate at which N_2_ is injected into the mixing chamber is very fast, so the process can be approximated as adiabatic, and the effects of outlet pressure loss and N_2_ quality can be ignored. Therefore, the mass flow rate of N_2_ can be determined according to the adiabatic flow theory[18].

$$G_{g}=\eta \frac{\pi d_{g}^{2}}{4}\sqrt{\frac{‐2\kappa }{\kappa -1}P_{g}\rho_{g}[{\left(\frac{P_{h}}{P_{g}}\right)}^{\frac{2}{\kappa }}-{\left(\frac{P_{h}}{P_{g}}\right)}^{\frac{\kappa +1}{\kappa }}]}$$ (1)

Where *G*_g_ is the mass flow rate of gas (*kg*/*s*); *P*_g_ and *ρ*_*g*_ are the gas pressure and gas density in the gas injection hole (*Pa*, *kg*/*m*^3^); *d*_g_ is the diameter of the gas injection hole (*m*); *P*_*h*_ is the pressure of the mixing chamber (*Pa*); *κ* is the N_2_ adiabatic index, and its value is generally 1.41; *η* is the flow coefficient of the gas injection hole, and its value is generally 0.8–0.9. It can be inferred from Eq (1) that the N_2_ flow rate is related not only to the gas supply parameters and pore structure, but also to the mixing chamber pressure.

When the gas-liquid two-phase flow atomising nozzle is working, the inhibitor water solution enters the gas-liquid mixing chamber from the liquid injection hole, and the pressure loss is composed of the frictional and local resistances. Owing to the short length of the section, the frictional resistance is negligible. The process of the inhibitor water solution flowing into the mixing chamber through the liquid injection hole is a sudden expansion flow, which will generate a large amount of local resistance loss in this region. The mass flow expression of the inhibitor water solution in the nozzle is

$$G_{w}=\rho_{w}2\sqrt{\frac{P_{w}-P_{h}}{\rho_{w}}}\pi {\left(\frac{d_{w}}{2}\right)}^{2}=\pi d_{w}^{2}\sqrt{\frac{\left(P_{w}-P_{h}\right)\rho_{w}}{4}}$$ (2)

Where *G*_w_ is the mass flow rate of water solution (*kg*/*s*); *P*_w_ and *ρ*_*w*_ are the liquid pressure and liquid density in the liquid injection hole (*Pa*, *kg*/*m*^3^); *d*_w_ is the diameter of the liquid injection hole (*m*). Normally, the volumetric flow of N_2_ is much larger than that of the inhibitor water solution, and thus, the pressure in the mixing chamber *P*_*h*_ is primarily affected by the gas pressure *P*_*g*_. According to Eq (2), the main factors that influence the mass flow of the inhibitor water solution include the diameter of the liquid injection hole and the operating parameters (pressure of the gas and inhibitor water solution). The mass flow of the inhibitor water solution is proportional to the square of the liquid injection hole diameter and 0.5 times the pressure difference (inhibitor water solution supply pressure and mixing chamber pressure) before and after the liquid injection hole.

The mixture of N_2_ and inhibitor-water mist in the mixing chamber of the nozzle forms a gas-liquid two-phase flow, and it is discharged from the outlet of the mixing chamber to the environment. The local resistance loss produced by the gas-liquid two-phase flow at the outlet of the mixing chamber can be calculated as the local resistance of the two-phase flow sudden expansion joint proposed by Romie[19].

$$\Delta P_{E}=P_{h}-P_{a}=\frac{v_{mh}^{2}{\left(1-B\right)}^{2}}{2\rho_{w}}\left[1+\chi \left(\frac{\rho_{w}}{\rho_{gh}}-1\right)\right]$$ (3)

Where Δ*P*_*E*_ is the local resistance loss of the mixing chamber outlet (*Pa*); *P*_*a*_ is the pressure of the environment (*Pa*); *ρ*_*gh*_ is the density at the mixing chamber (*kg*/*m*^3^); *B* is the area ratio between the upstream and downstream sections of the sudden expansion outlet, and for the two-phase flow in the mixing chamber, *B* = 0; *χ* is the mass gas content, i.e., dryness, *χ* = *G*_*g*_/*G*_*w*_; and *v*_*mh*_ is mass velocity of the two-phase, $v_{mh}=G_{h}/\left(\frac{1}{4}\pi d_{h}^{2}\right)$, *kg*/(*m*^2^∙*s*). Substituting the relevant parameters into Eq (3),

$$P_{h}-P_{a}=\frac{1}{2\rho_{w}}[\frac{4G_{h}}{\pi d_{h}^{2}}{]}^{2}\left[1+\frac{G_{g}}{G_{h}}\left(\frac{\rho_{w}}{\rho_{gh}}-1\right)\right]$$ (4)

For a constant flow, according to the conservation of mass,

$$G_{h}=G_{g}+G_{w}$$ (5)

Eqs (1)–(5) constitute the equations for solving the gas and liquid mass flow rate of the gas-atomising nozzle. According to the structural parameters of the nozzle and the known operating parameters, the mass flow rate of N_2_ and inhibitor-water mist can be obtained under the corresponding working conditions.

As early as in 1939, Nukiyama[20] carried out a research on the atomisation of the flat-hole type air-blast nozzle. On this basis, the calculation equation of the atomisation particle size of the two-phase flow nozzle is derived.

$$D_{SMD}=585\left(\frac{1000}{v_{r}}\sqrt{\frac{\sigma_{w}}{\rho_{w}}}\right)+597{\left(\frac{\mu_{w}}{100\sqrt{\sigma_{w}\rho_{w}}}\right)}^{0.45}{\left(1000\frac{Q_{w}}{Q_{g}}\right)}^{1.5}$$ (6)

In this equation, *D*_*SMD*_ is the Sauter mean diameter (SMD) (*μm*); *σ*_*w*_ is the surface tension coefficient of the liquid (*N*/*m*); *μ*_*w*_ is the coefficient of viscosity of the liquid (*Pa*∙*s*); *v*_*r*_ is the relative velocity of gas and liquid in the mixing chamber (*m*/*s*), $v_{r}=\sqrt{{\left(v_{g2}\sin\alpha \right)}^{2}+{\left(v_{g2}\cos\alpha -v_{w2}\right)}^{2}}$; *Q*_*g*_, *Q*_*w*_ are the volume flow of gas and liquid (*m*^3^/*s*), respectively; *α* is the velocity angle of gas and liquid.

#### Experimental verification of NIWM equipment operating parameters

**Fig 3:**
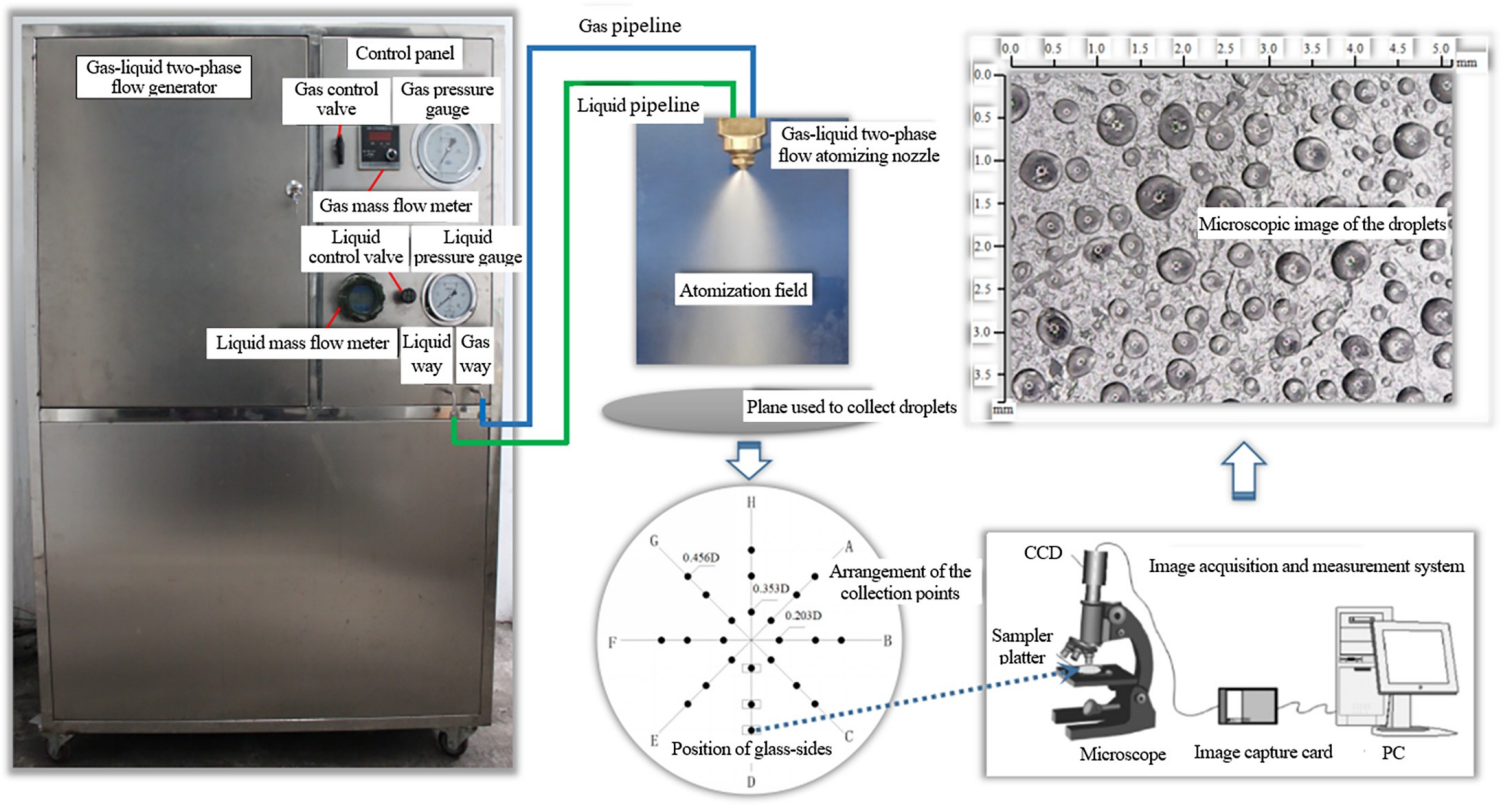
Experimental system for operating parameters of NIWM fire prevention and extinguishing equipment.

In order to verify the theoretical derivation of operating parameters of NIWM equipment, the experiment shown in Fig 3 was carried out. The NIWM two-phase flow generator system comprises two parts, namely, liquid and gas supply systems. To better study the atomisation characteristics of the equipment, many parameters are used to measure the spray quality of the equipment, including droplet size, which is particularly important. In the current technical research, the methods for droplet size measurement include the phase Doppler particle analyser (PDPA)[21] and the Malvin particle analyser[22]. The particle size measured by the instruments used in these two measurement methods has high precision, but the instrument structure is complicated, they are expensive, and there are many influencing factors in the measurement. The charge-coupled device (CCD) image sensing technology[23] has been developed rapidly due to its advantages of low cost, simple operation, high running speed, and high precision. Therefore, the CCD image sensing technology was used to study the technical parameters of the NIWM fire prevention and extinguishing equipment. In the experiment, we first determined the diameter D of the atomisation field at a point located 1 m under the nozzle, prepared a number of glass-slides coated with petrolatum, and placed the glass-slides at the 24 placement points shown in the droplet collection plane in Fig 3. After that, the NIWM equipment was utilised to perform image collection and size measurement of the droplets.

**Fig 4:**
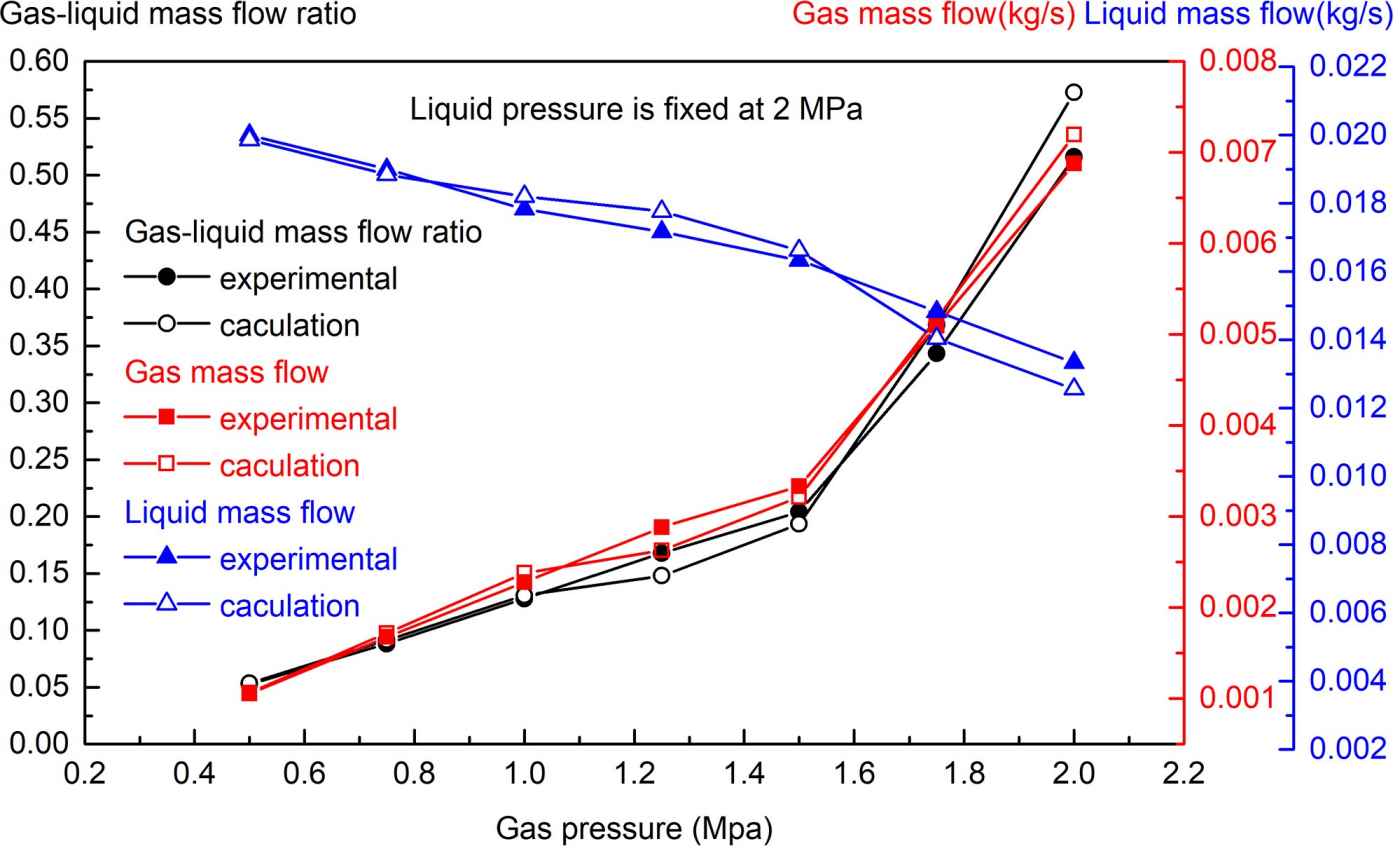
Effect of gas pressure change on gas and liquid mass flow (water pressure is 2 MPa, gas pressure is 0.5–2 MPa).

**Fig 5:**
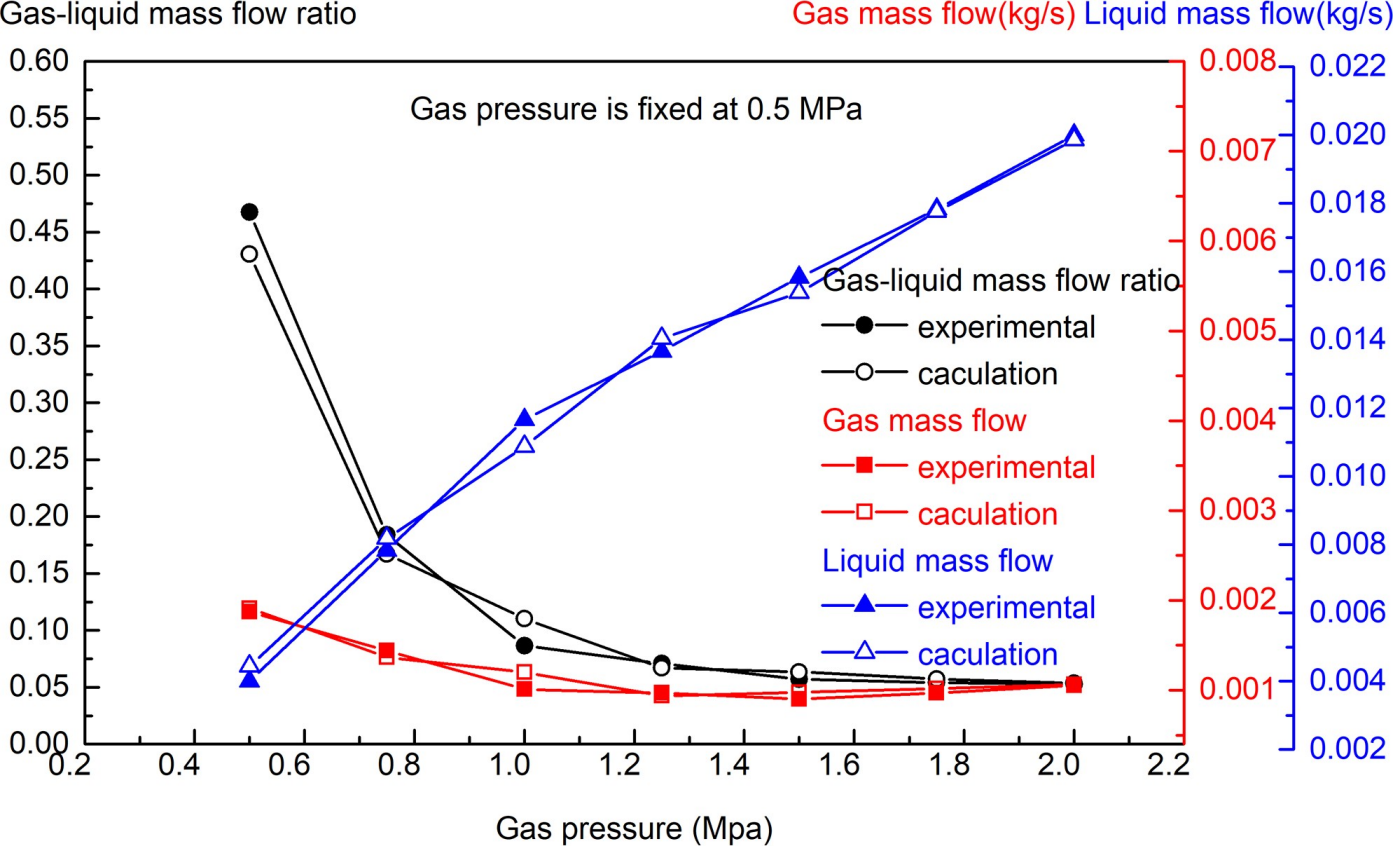
Effect of water pressure change on gas and liquid mass flow (water pressure is 0.5–2 MPa, gas pressure is 0.5 MPa).

**Fig 6:**
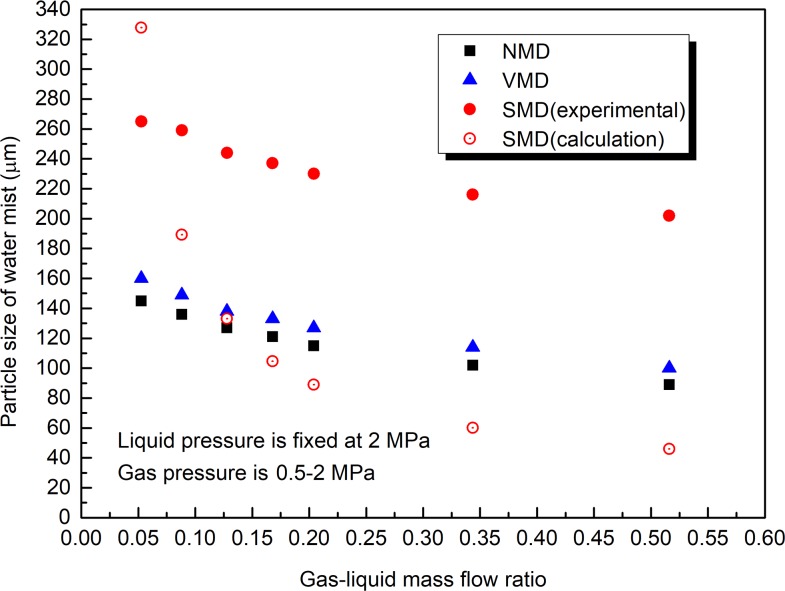
Effect of gas–liquid mass flow ratio on water mist particle size (water pressure is 2 MPa, gas pressure is 0.5–2 MPa).

**Fig 7:**
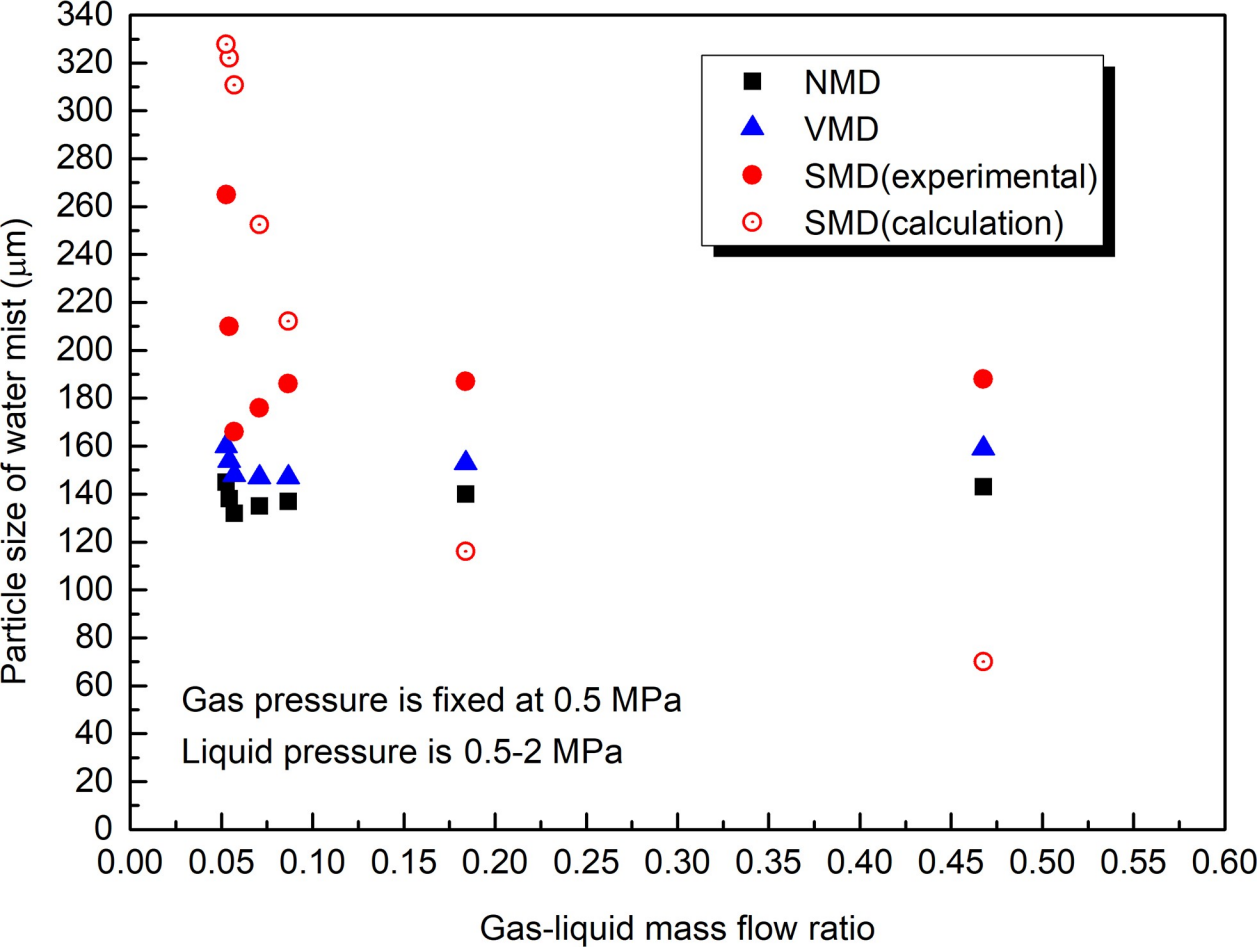
Effect of gas-liquid mass flow ratio on water mist particle size (water pressure is 0.5–2 MPa, gas pressure is 0.5 MPa).

Two groups of experiments were designed to study the atomisation law. In one group of the experiments, the gas pressure was changed while the water pressure was kept constant, whereas in the other group of experiments, the water pressure was changed while the gas pressure was kept constant. In the experimental stage, the maximum gas pressure was 2.5 MPa. The equipment could only produce the gas-liquid two-phase flow if the water pressure was greater than the gas pressure in mixing chamber (this phenomenon was consistent with the theoretical derivation of formula (2)). *D*_*SMD*_ is equivalent to the ratio of the diameter of the spherical particle having the volume of all particles to its surface area, $D_{SMD}=\left(\int_{D_{\min}}^{D_{\max}}D_{i}^{3}dN\right)/\left(\int_{D_{\min}}^{D_{\max}}D_{i}^{2}dN\right)$. In addition, the other commonly used indicators for measuring droplet size include the number median diameter (NMD) and volume median diameter (VMD). The droplet size under different parameters are shown in Figs 4–7.

As shown in Fig 4, when the liquid pressure is fixed at 2 MPa and the gas pressure is changed within the range of 0.5–2 MPa, with the increase in gas pressure, the mass flow of gas increases, the liquid flow decreases, and the gas-liquid mass flow ratio increases. The change in gas pressure affects the flow of gas and liquid, and the effect is substantial. As shown in Fig 5, when the gas pressure is fixed at 0.5 MPa and the water pressure is changed within the range of 0.5–2 MPa, with the increase of water pressure, the mass flow of water increases, the mass flow of gas remains basically unchanged, and the gas-liquid mass flow ratio decreases. The change in liquid pressure has a significant effect on the liquid mass flow rate, but it has little effect on the gas mass flow rate. The experimental results in Figs 4 and 5 are basically consistent with the theoretical calculation results of Eqs (1)–(5).

As shown in Fig 6, when the liquid pressure is fixed at 2 MPa and the gas pressure is changed within the range of 0.5–2 MPa, with the increase in the gas-liquid mass flow ratio, the NMD, VMD, and SMD decrease. The gas-liquid mass flow ratio has a significant effect on the water mist particle size. As shown in Fig 7, when the gas pressure is fixed at 0.5 MPa and the water pressure is changed within the range of 0.5–2 MPa, with the increase in the gas-liquid mass flow ratio, SMD decreases first and then remains unchanged, while NMD and VMD remain unchanged. The increase in the gas-liquid mass flow ratio can reduce the water mist particle size, but there is a certain threshold. After the gas-liquid mass flow rate ratio is greater than the threshold, the water mist particle size is no longer significantly affected. From Figs 6 and 7, it is known that the water mist particle size when the gas pressure is fixed at 0.5 MPa and the water pressure is changed within the range of 0.5–2 MPa is significantly larger than that when the liquid pressure is fixed at 2 MPa and the gas pressure is changed within the range of 0.5–2 MPa. The main reason is that the water mist particle size is also affected by the gas and liquid velocity (as shown in Eq (6)). In general, the particle size of the droplet decreases as the gas velocity increases, and it increases as the liquid velocity increases. The experimental results in Figs 6 and 7 are basically consistent with the theoretical calculation results of Eq (6).

In summary, during the implementation of the NIWM goaf fire prevention and extinguishing equipment, after the equipment is designed, the flow rate of the N_2_ and inhibitor-water solution and the particle size of the inhibitor water mist can be controlled by adjusting the pressure of the N_2_ and inhibitor water solution, so as to achieve the best goaf fire prevention and extinguishing effect. The experimental results are consistent with the theoretical derivation, which can be used as a theoretical guidance for using the NIWM fire prevention and extinguishing technology.

## Experimental research on NIWM fire prevention and extinguishing

### Experimental equipment and scheme

**Fig 8:**
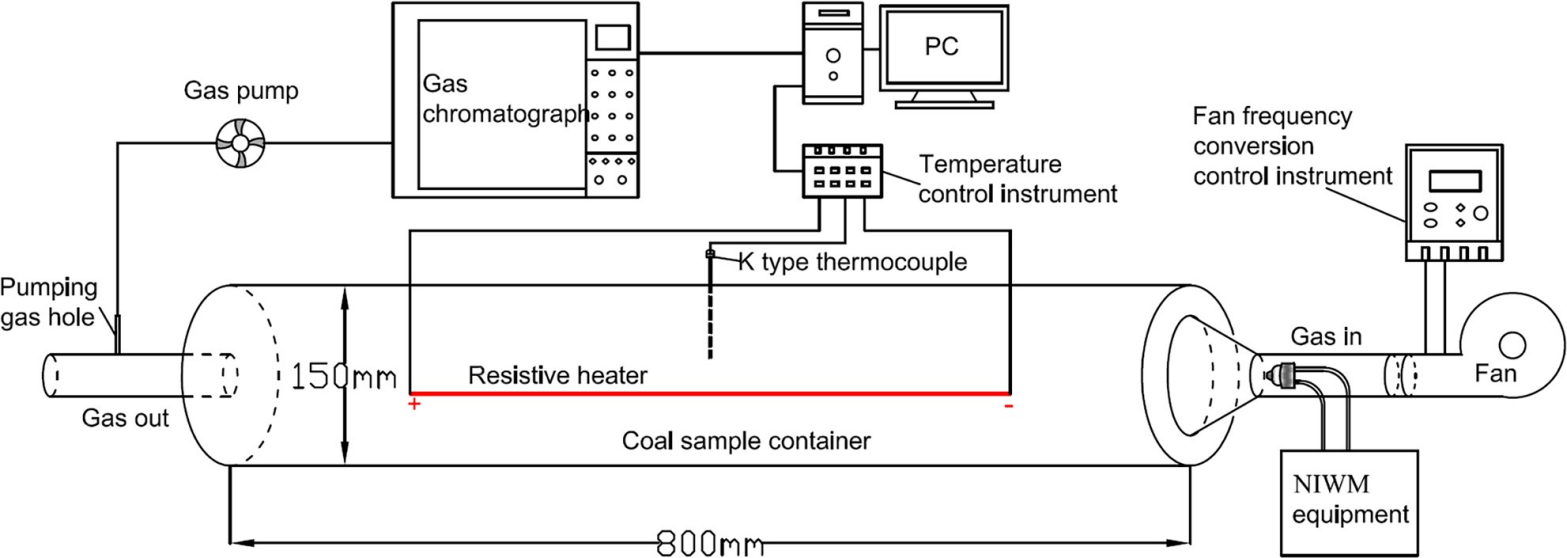
Experimental setup of NIWM fire prevention and extinguishing.

In general, the oxidative exothermic of coal in low temperature stage is the direct cause of spontaneous combustion of coal[24–26]. In previous studies, the control of spontaneous combustion in mine goaf includes two parts: inhibit low temperature oxidation of coal[27–29] and extinguish high temperature combustion of coal[30–33]. Thus, the experiment of inhibiting low temperature oxidation of coal by NIWM and extinguishing high temperature combustion of coal by NIWM were carried out, respectively. The experimental setup is shown in Fig 8. In the experiments, the coal samples were placed in the container with resistive heater inside. The change of ambient temperature of coal-oxygen reaction was realized by heating resistive heater. The air for coal-oxygen reaction was provided by the fan, and the air volume was controlled by the fan frequency conversion control instrument. Gases produced by coal-oxygen reaction were pumped to gas chromatography for detection and analysis. The gas-liquid two-phase flow atomising nozzle of the NIWM equipment was installed at the gas inlet. N_2_ and water mist containing inhibitor were sprayed into the container to study the effect of NIWM on coal-oxygen reaction. In the experiments, fresh raw coal sample of 8# coal seam of Xishan Coalfield, China was transported to the laboratory with plastic wrap. The coal sample was milled in laboratory under isolated air condition and sieved into the particle size of 5–50mm. Properties of the coal sample are shown in Table 2.

**Table 2:** Properties of the coal sample.

proximate analysis (W/%)			ultimate analysis (W_daf_/%)				
M_ad_	A_ad_	V_daf_	C	H	O	N	S
0.96	6.52	15.13	88.10	3.83	4.82	1.12	2.13

#### Inhibiting low temperature oxidation of coal by NIWM

In the the experiment of inhibiting low temperature oxidation of coal by NIWM, five groups of experiments were set up. The first group only ventilated air (L-1: Air), the second group added the injection of N_2_ (L-2: Air+N_2_). The third group added the injection of N_2_-water mist (L-3: Air+N_2_-water mist). The fourth group added the injection of N_2_-inhibitor-water mist (L-4: Air+N_2_-inhibitor-water mist). In the fifth group, the inhibitor-water liquid was provided by a single-phase pressure nozzle, N_2_ and inhibitor-water liquid were injected into the container independently (L-5: Air+N_2_+inhibitor-water liquid). In each group, the temperature range was 30–100°C, the heating rate was 0.5°C/min, the coal mass was 10kg, and the air flow rate was 30L/min. In L-2, L-3, L-4 and L-5 groups, the injection of N_2_ continued throughout the experiment, and the N_2_ pressure and flow rate were 0.5MPa and 87L/min, respectively. In L-3, L-4 and L-5 groups, the water pressure and flow rate were 0.5MPa and 0.24L/min, respectively. According to the results of the above research on NIWM equipment, it can be known that the SMD of water mist produced in L-3 and L-4 groups under the experimental conditions was 188 μm. The water drop produced in L-5 group under the experimental conditions was >1000 μm, thus it was defined as inhibitor-water liquid. 1L water mist, water mist containing inhibitor and water liquid containing inhibitor were injected before the experiments in L-3, L-4 and L-5 groups, respectively. The inhibitor used in L-4 and L-5 groups was CaCl_2_, and the mass of it was 0.25kg. Water mist in L-3 and L-4 groups was obtained by N_2_ atomization. The inhibition effect of NIWM on coal-oxygen reaction was studied by analyzing the formation of CO and CO_2_ at different temperatures in the five groups of experiments.

#### Extinguishing high temperature combustion of coal by NIWM

In the the experiment of extinguishing high temperature combustion of coal by NIWM, the coal sample was first ignited by continuous heating, and the heating was stopped after the occurrence of open fire, and then the NIWM fire extinguishing experiment was carried out. Five groups of comparative experiments were also set up in this stage. The first group was free combustion under ventilation (H-1: Air). The second group was fire extinguishing by continuously injecting N_2_ (H-2: Air+N_2_). The third group was fire extinguishing by continuously injecting N_2_-water mist (H-3: Air+ N_2_-water mist). The fourth group was fire extinguishing by continuously injecting N_2_-inhibitor-water mist (H-4: Air+N_2_-inhibitor-water mist). In the fifth group, the inhibitor-water liquid was provided by a single-phase pressure nozzle, N_2_ and inhibitor-water liquid were injected into the container independently (H-5: Air+N_2_+inhibitor-water liquid). In each group, the coal mass was 10kg, and the air flow rate was 30L/min. During the whole fire extinguishing experiment, the N_2_ pressure and flow rate were 0.5MPa and 87L/min, and the water pressure and flow rate were 0.5MPa and 0.24L/min. The SMD of water mist produced in H-3 and H-4 groups under the experimental conditions was 188μm. The water drop produced in H-5 group under the experimental conditions was >1000μm. In the H-4 and H-5 groups, the concentration of CaCl_2_ in the inhibitor water solution was 20%. Each group lasted for 30 minutes. The extinguishing effect of NIWM on coal combustion was studied by analyzing the change of temperature and the formation of CO and CO_2_ at different times in the five groups of experiments.

## Results and discussions

### Inhibiting low temperature oxidation of coal by NIWM

**Fig 9:**
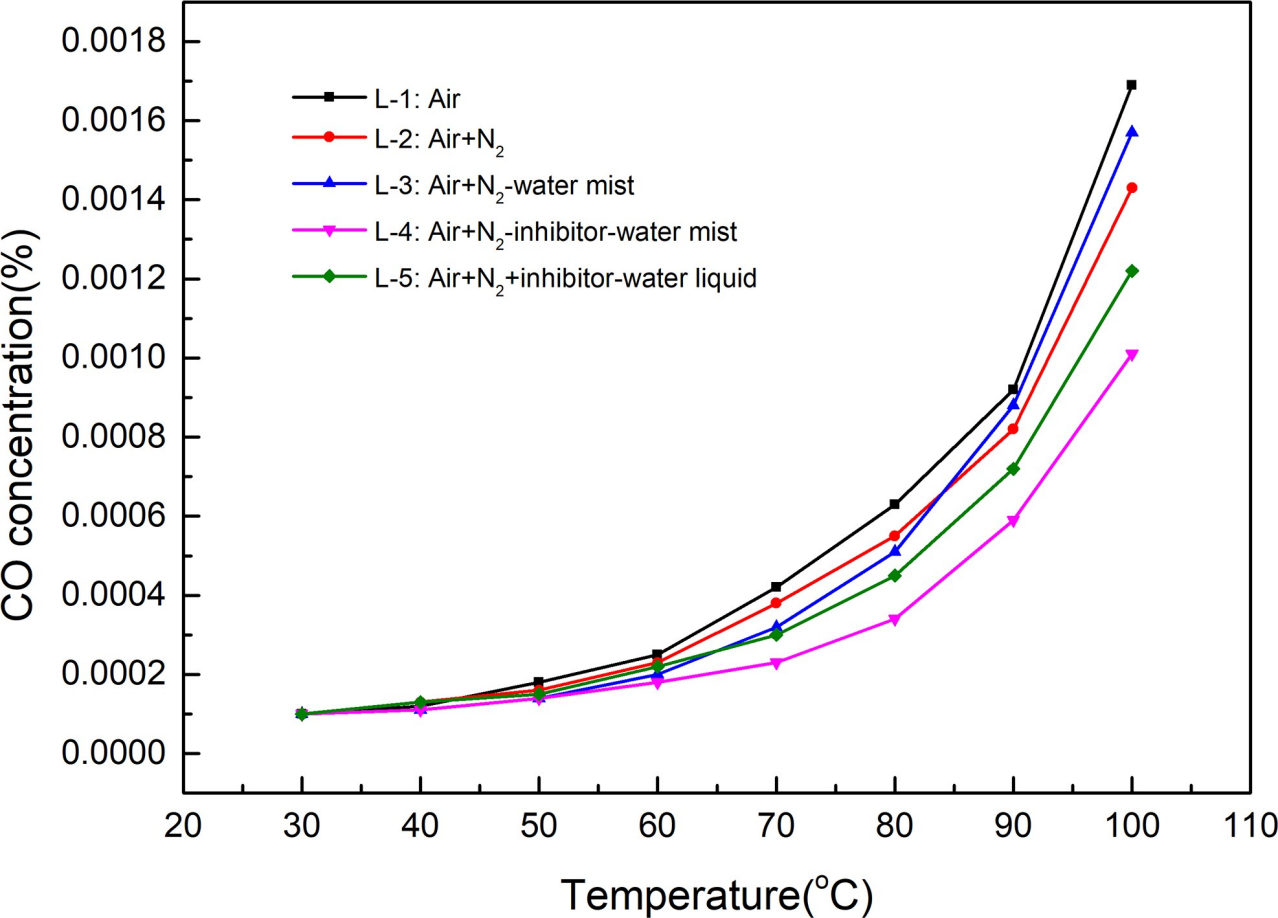
Inhibition effect of NIWM on CO generated by coal-oxygen reaction.

**Fig 10:**
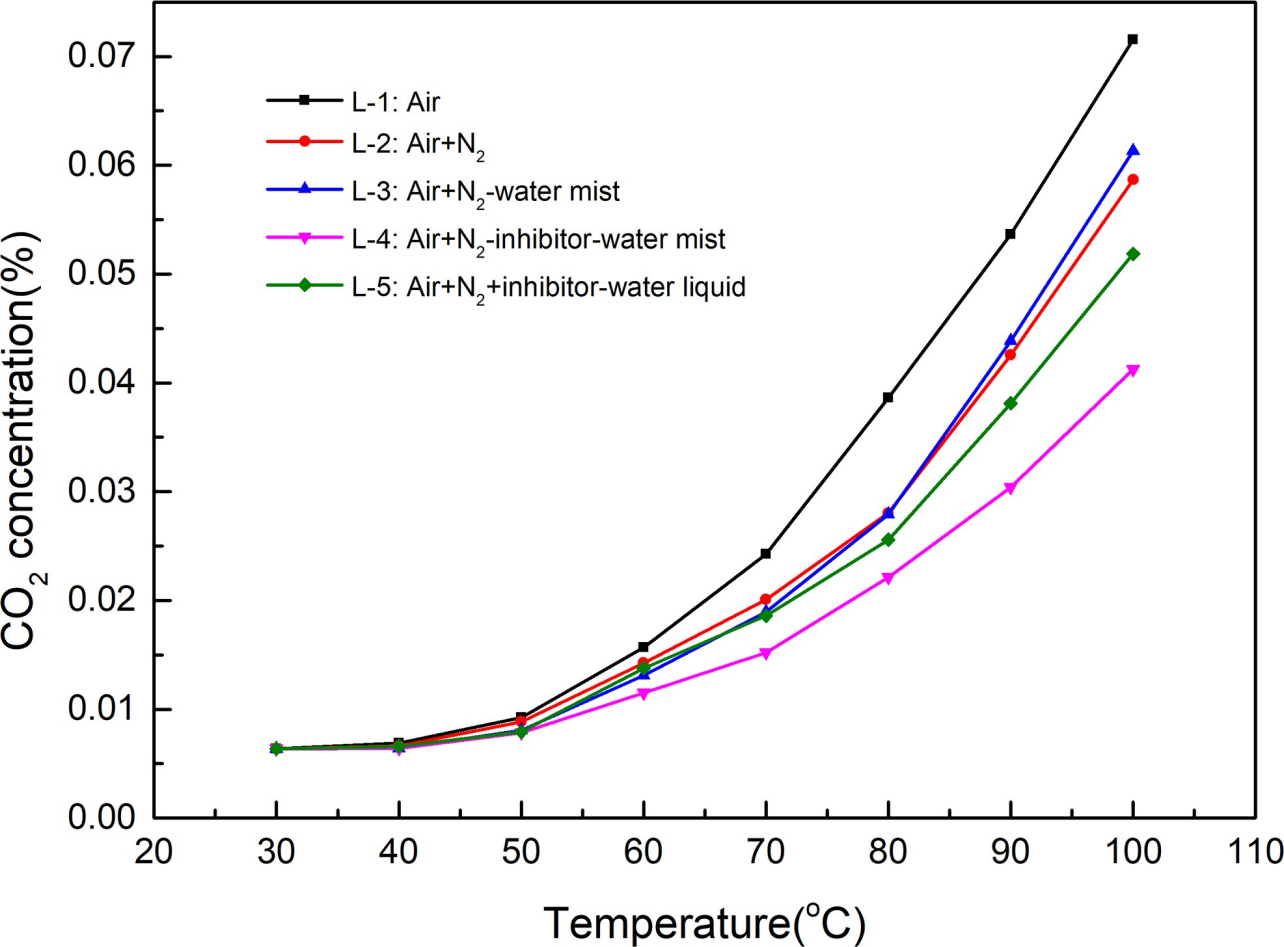
Inhibition effect of NIWM on CO_2_ generated by coal-oxygen reaction.

In terms of self-heating of coal, the low temperature region is an important stage of coal spontaneous combustion and has always been the focus of research[28,29,34–40]. Generally, the three processes are believed to occur in the low temperature coal-oxygen reaction[41]: (i) physical adsorption of oxygen; (ii) chemical adsorption which leads to the formation of coal-oxygen complexes and oxygenated carbon compounds; and (iii) oxidation in which the coal and oxygen react with the release of gaseous products, typically CO, CO_2_, and H_2_O. The amount of the produced CO and CO_2_ can reflect the extent of the coal-oxygen reaction. The changes of CO and CO_2_ in the experiments of inhibiting low temperature oxidation of coal by NIWM are shown in Figs 9 and 10.

As shown in Figs 9 and 10, the injection of N_2_, N_2_-water mist, N_2_-inhibitor-water mist and N_2_+inhibitor-water liquid all reduced the production of CO and CO_2_. Compared with N_2_, the inhibition effect of N_2_-water mist on coal-oxygen reaction was greater than that of N_2_ in the early stage of heating, but with the increase of temperature, the inhibition effect was less than that of N_2_. The inhibition effect of N_2_-inhibitor-water mist was obviously greater than that of N_2_, N_2_-water mist and N_2_+inhibitor-water liquid. O_2_ concentration is an important factor in the three elements of material combustion[42]. The injection of a large amount of N_2_ can rapidly reduce the concentration of O_2_. The O_2_ concentration in L-2, L-3, L-4 and L-5 groups was reduced from 21% to 5.38% by the injection of N_2_. N_2_ partially replaces O_2_ and enters the fracture surface of the coal, and then it adsorbs on the microscopic surface of the coal. As a result, the amount of O_2_ adsorbed on the surface of the coal is reduced, and the oxidation of the coal is greatly slowed or suppressed[43]. In the early stage of heating, the injection of water mist (L-3 group) can hinder O_2_ migration in coal fissures and pores, thus the coal-oxygen reaction is inhibited. However, with the increase of temperature, water mist in coal fissures and pores begins to evaporate gradually. Large amount of evaporation of water mist will increase the fissure and pore channels of coal, thereby increasing the adsorption capacity of coal to O_2_ and promoting the coal-oxygen reaction[44]. Therefore, when water mist is used alone to inhibit spontaneous combustion of coal, the water content has a dual effect of inhibiting and promoting. Comparing L-3 and L-4 groups, it can be known that the addition of inhibitor can effectively inhibit the coal-oxygen reaction. Inhibitor contains a certain amount of moisture, and some inhibitors can absorb the moisture in the air. The presence of moisture inhibits the rise of coal temperature[45]. The thermal conductivity of inhibitor is better than that of the coal body, which is conducive to heat dissipation. At the same time, the evaporation of moisture in the inhibitor absorbs a significant amount of heat. In the process of free radical chain reaction of coal, the inhibitor is involved as a chemical component that produces some stable chain rings[46]. This increases the activation energy of the reaction between the free radicals of the coal surface and the O_2_, and slows down and inhibits the coal surface activity, thus preventing the spontaneous combustion of coal. Comparing L-4 and L-5 groups, it can be known that the spraying form of inhibitor has significant effect on inhibiting coal-oxygen reaction. The inhibitor sprayed in the form of water mist has better diffusivity, can cover the coal body more evenly, and has better inhibition effect.

Compared with single fire prevention measures, N_2_-inhibitor-water mist can more effectively inhibit coal-oxygen reaction. N_2_ can effectively atomise and carry inhibitor-water mist. This technology achieved three-dimensional coverage of the coal by the N_2_, inhibitor and water mist.

#### Extinguishing high temperature combustion of coal by NIWM

**Fig 11:**
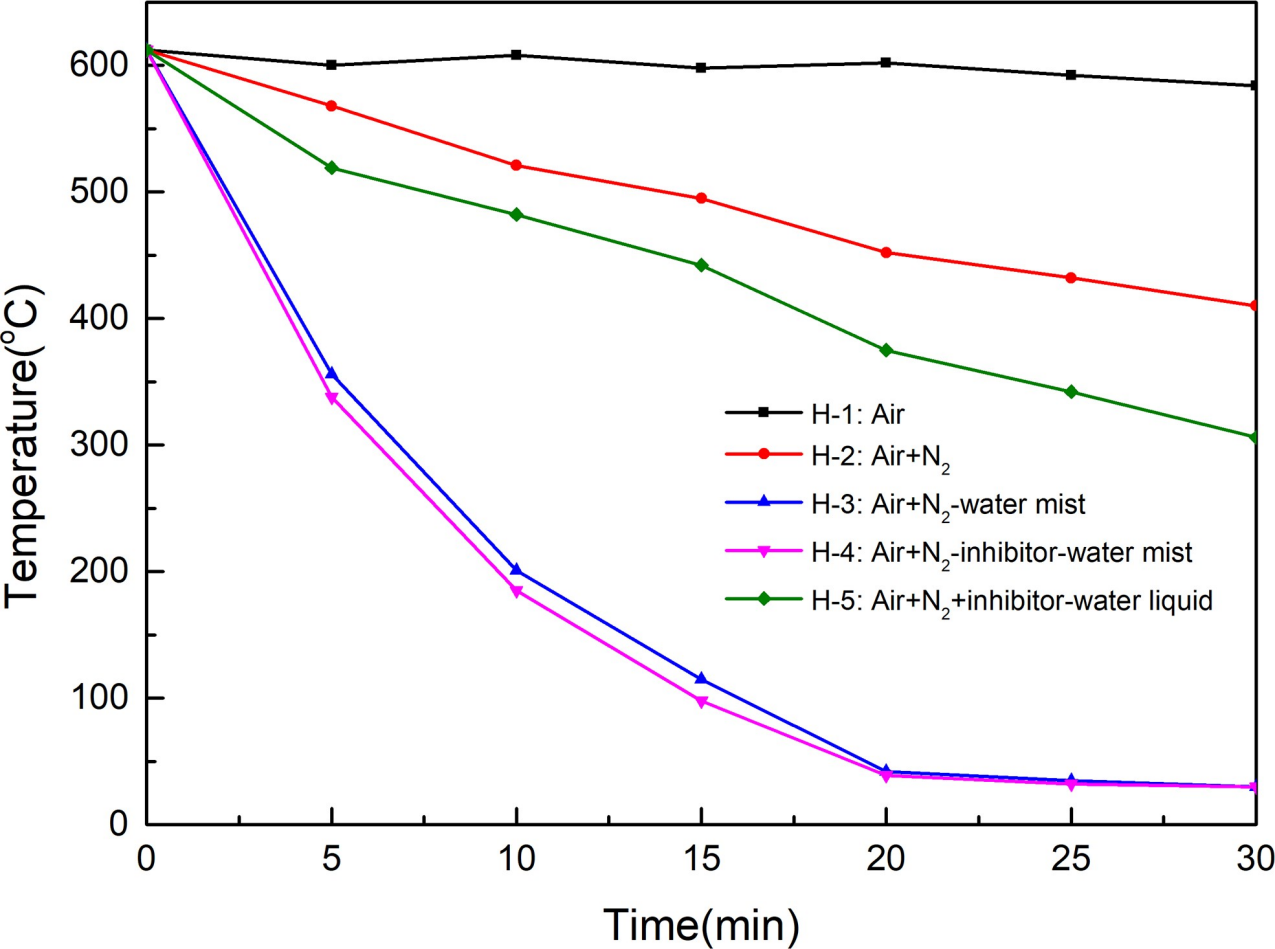
Extinguish effect of NIWM on temperature of coal combustion.

**Fig 12:**
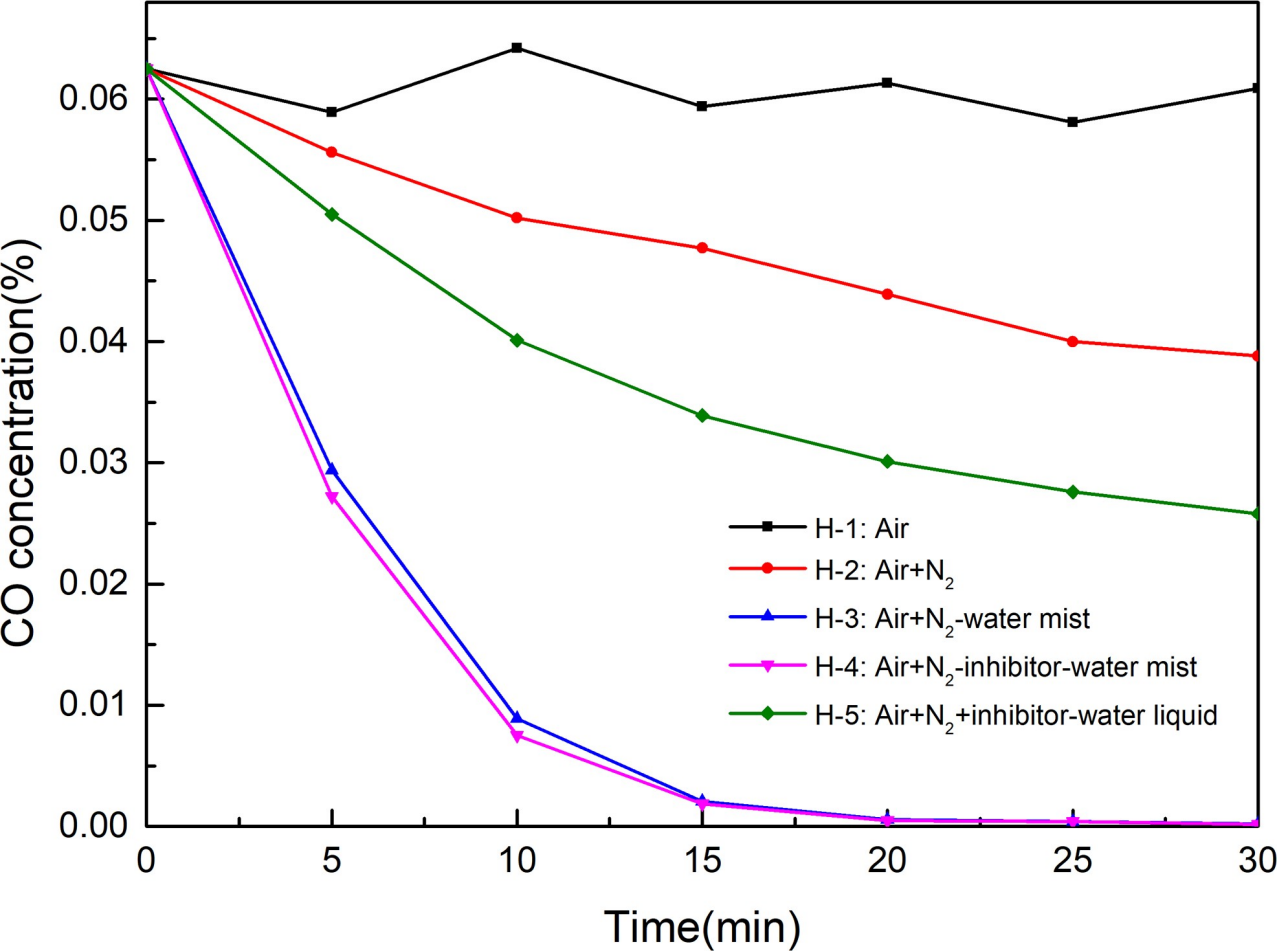
Extinguish effect of NIWM on CO of coal combustion.

**Fig 13:**
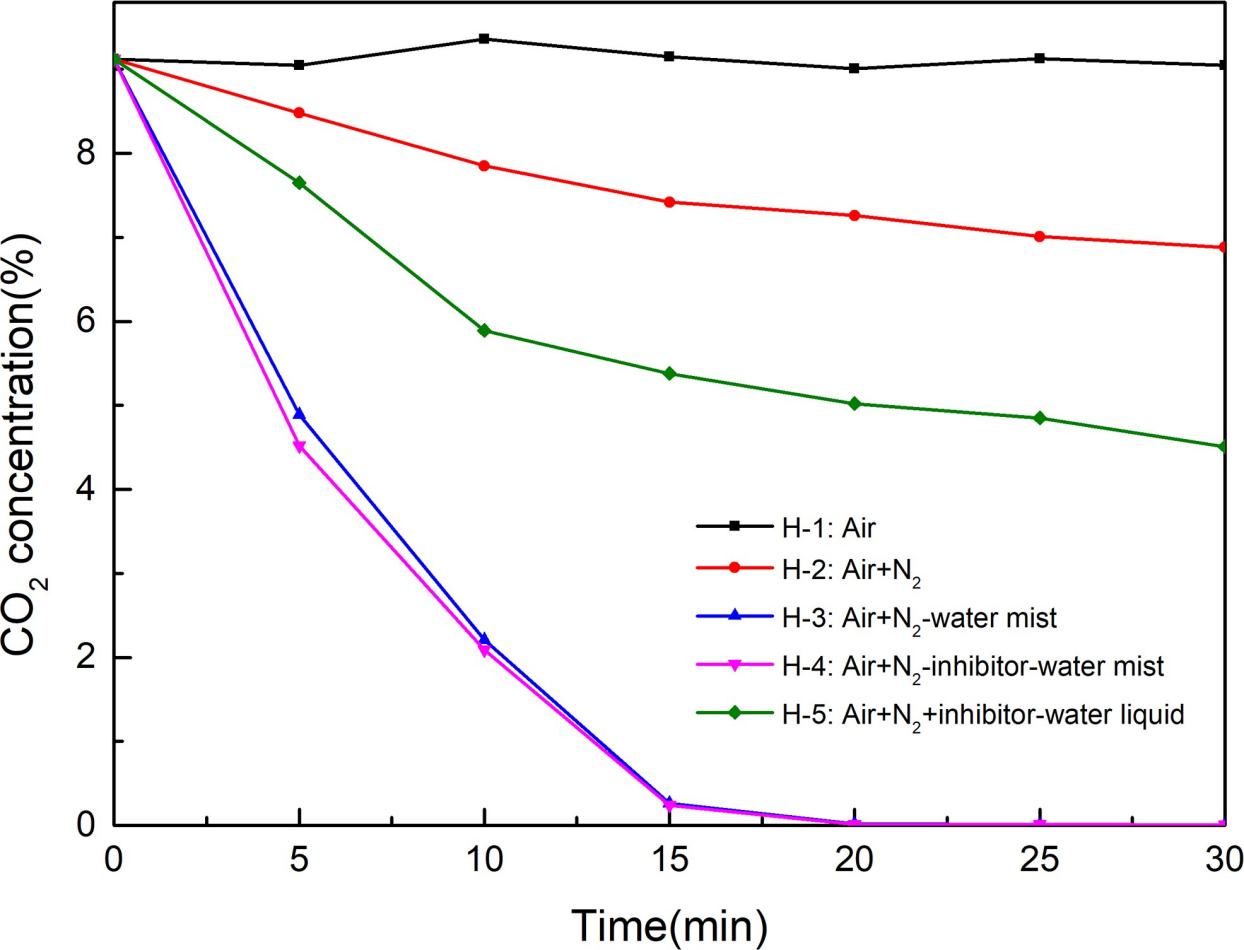
Extinguish effect of NIWM on CO_2_ of coal combustion.

In the experiments of extinguishing high temperature combustion of coal, the time at which NIWM was injected is used as the starting point of time. The changes of temperature, CO and CO_2_ with time under different conditions are shown in Figs 11–13. The injection of N_2_, N_2_-water mist, N_2_-inhibitor-water mist and N_2_+inhibitor-water liquid all reduced the temperature and the production of CO and CO_2_. The cooling process of N_2_ was very slow, and after 30 minutes of application, the temperature inside the container was still 410°C. The main function of N_2_ is to reduce the concentration of O_2_, but coal-oxygen reaction can still be carried out under low O_2_ concentration, so it is difficult to extinguish mine fire in a short time simply by using N_2_. This conclusion is consistent with the field effect of N_2_ fire extinguishing in recent years[30–33]. Both the injection of N_2_-water mist (H-3 group) and N_2_-inhibitor-water mist (H-4 group) rapidly reduced the temperature in the container. In the experiments of H-3 and H-4 groups, when the experiment lasted for 20 minutes, the burned coal was completely extinguished. Comparing H-3 and H-4 groups, in can be known that the fire extinguishing effect was not significantly improved after the injection of inhibitor. Therefore, water mist played a leading role in the fire extinguishing process. However, the fire extinguishing effect of N_2_+inhibitor-water liquid (H-5 group) was far less than that of N_2_-water mist and N_2_-inhibitor-water mist. This phenomenon fully demonstrated that water mist has the characteristics of good diffusibility and rapid cooling.

Water mist is a new type of coal mine fire extinguishing technology. It can control, suppress, and extinguish fire through the multiple effects of cooling, asphyxiating, isolating heat radiation, and moistening. It has the dual role of water spray and gas extinguishment. Compared with traditional fire extinguishing technologies, it has the advantages of good fire prevention, low water consumption, no pollution, and the use of a nozzle that is not easy to plug[47–49]. A large amount of water mist accompanied by air leakage and N_2_ entering the goaf will vaporise rapidly after encountering the high-temperature coal rock mass. In the process of vaporisation, a large amount of heat will be absorbed, which can rapidly reduce the temperature of the goaf. In the process of vaporisation of the water mist, its volume expands rapidly, which forms a barrier in the fire area, and then prevents the consumption of fresh air, reduces the volume fraction of O_2_ in the fire area, and inhibits the burning of the coal. The water mist entering the fire area can rapidly envelop the combustibles, flames, and smoke plumes, thus blocking the transfer of flame heat radiation to a certain extent and effectively preventing the spread of the flame. The water mist entering the goaf reaches the surface of the coal, makes the coal fully moist, and prevents further generation of volatile flammable gas. Thus, the purpose of extinguishing the fire and preventing its spread is realised.

The effect of N_2_-inhibitor-water mist on extinguishing combustion coal is significantly different from that of inhibiting oxygen reaction at low temperature. In the process of extinguishing combustion coal, the effect of inhibitor is not significant. Water mist could reduce the temperature and the production of CO and CO_2_ of the fire area in a short time. Although the fire extinguishing effect of N_2_ is slow, it can be used as spray source and carrier of water mist. Therefore, the N_2_-water mist can be used as an effective fire extinguishing technology in goaf.

## Design of field process flow

The experiment results showed that NIWM has better effect on inhibiting spontaneous combustion and extinguishing combustion of coal. In different stages of coal-oxygen reaction, N_2_, inhibitor and water mist play very different role in controlling the process of coal-oxygen reaction, which was not simple accumulation of the three. N_2_ can inhibit the coal-oxygen reaction in the low temperature oxidation stage and combustion stage, but the cooling effect is slow. Inhibitor can only inhibit the coal oxygen reaction in the low temperature oxidation stage. Water mist has the dual effects of inhibiting and promoting the coal-oxygen reaction in the low temperature oxidation stage and can rapidly reduce the coal-oxygen reaction temperature in the high temperature stage. In the low temperature oxidation stage, the effective carrying effect of water mist on inhibitor is mainly used, while in the high temperature combustion stage, water mist is used for good diffusion and cooling effect.

**Fig 14:**
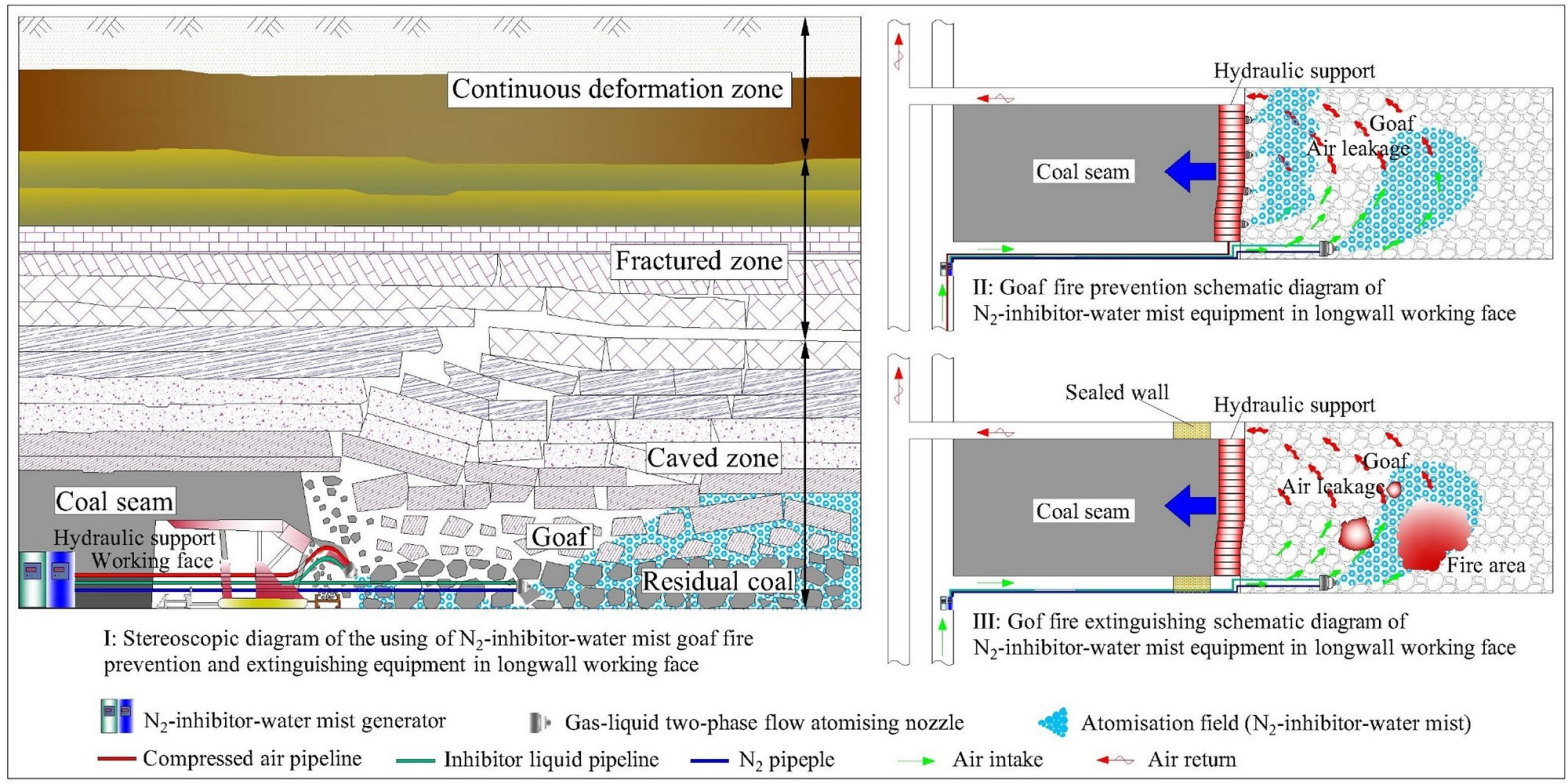
Onsite arrangement diagram of the NIWM fire prevention and extinguishing equipment in the goaf of a longwall working face.

Based on the experimental results above, the onsite arrangement of the NIWM fire prevention and extinguishing equipment in the goaf area of a longwall working face is designed (shown in Fig 14). In the low temperature oxidation stage, N_2_ and inhibitor of NIWM are mainly used to inhibit coal oxidation, and the inhibitor should be sprayed evenly on the coal body to the maximum extent. Thus, the application of the NIWM equipment can be divided in two parts. 1) Initial spraying on the rear of the working face hydraulic support. The spraying point is near the working surface and is not deep into the goaf. Thus, in order to ensure that both the O_2_ concentration at the working face and the cost are reduced, a compressed air pipeline laid underground is used as the gas source; the spraying method adopts multi-point automatic spraying, and the spraying step is one propulsion cycle. 2) Secondary diffusion spraying in the deep goaf. As time goes on, the inhibitor function is lost, and the hidden danger of spontaneous combustion in the goaf increases. The inhibitor–water mist is sent to the deep part of the goaf for secondary diffusion spraying by using the carriers of N_2_ and air leakage. The two-phase flow atomising nozzle of the NIWM generator is arranged in the deep part of the goaf in advance. In this way, long-distance transportation of the inhibitor-water mist is avoided, and it is directly diffused to the deep part of the goaf after being sprayed. Then, its full migration is realised under the common driving of air leakage and N_2_ in the goaf. In the high temperature combustion stage, N_2_ and water mist of NIWM are mainly used to extinguish coal combustion. When the fire occurs in goaf, the working face is first closed, and then a large amount of N_2_ and water mist are injected into the fire area through the two-phase flow atomising nozzles behind the hydraulic support and inside the goaf.

## Conclusions

In this paper, the NIWM fire prevention and extinguishing technology was proposed, and the corresponding equipment was designed and manufactured. The theoretical equations of the operating parameters of the equipment were derived, and the correctness of the theoretical equations were verified by experiments. The experiment of inhibiting low temperature oxidation and extinguishing high temperature combustion of coal by the NIWM equipment were carried out.

Under the condition that both gas pressure and liquid pressure were 0.5–2MPa, the NIWM equipment produced the water mist with Sauter mean diameter (SMD) range of 166–265μm. Good atomisation effect was produced under low working pressure. The experimental results of the operating parameters of NIWM equipment were in agreement with the theoretical results. The theory of two-phase flow atomisation can be used as a theoretical guide for this technology.

In the experiment of inhibiting low temperature (30–100°C) oxidation of coal by NIWM, NIWM effectively inhibited the coal-oxygen reaction, and the effect of it was better than that of single material. When the gas pressure and water pressure were both 0.5 MPa, the inhibitor water mist (SMD = 188 μm) was transported long distance in the container and evenly covered on the coal body. Water mist inhibited coal-oxygen reaction first and then promoted it. In the the experiment of extinguishing high temperature (600°C) burning coal by NIWM, N_2_ reduced the temperature of the container from 600°C to 410°C in 30 minutes, while N_2_-water mist extinguished the fire completely in 20 minutes. The addition of water mist solved the shortcoming of poor cooling effect of N_2_. Inhibitor (CaCl_2_) had little effect on fire extinguishing effect at high temperature. The cooling and extinguishing effect of water mist was better than that of water liquid. The experiment results showed that NIWM has better effect on inhibiting spontaneous combustion and extinguishing combustion of coal. In different stages of coal-oxygen reaction, N_2_, inhibitor and water mist play very different role in controlling the process of coal-oxygen reaction, which was not simple accumulation of the three. N_2_ can inhibit coal-oxygen reaction in the whole stage. Its function includes reducing the O_2_ concentration and acting as spray source and carrier of inhibitor/water mist. Inhibitor can only inhibit the coal oxygen reaction in the low temperature oxidation stage. Water mist has the dual effects of inhibiting and promoting the coal-oxygen reaction in the low temperature oxidation stage and can rapidly reduce the coal-oxygen reaction temperature in the high temperature stage. The combination of N_2_, inhibitor and water mist should be determined according to the state of the coal mine goaf fire.

On the basis of the research conclusions, the onsite arrangement diagram of the NIWM fire prevention and extinguishing equipment in the goaf was designed. The research is of great significance to the prevention and control of coal spontaneous combustion in goaf.
